# An Experimental Study on the Attribution of Personality Traits to Bullies and Targets in a Workplace Setting

**DOI:** 10.3389/fpsyg.2017.01045

**Published:** 2017-06-22

**Authors:** Ståle Pallesen, Morten B. Nielsen, Nils Magerøy, Cecilie S. Andreassen, Ståle Einarsen

**Affiliations:** ^1^Department of Psychosocial Science, University of BergenBergen, Norway; ^2^National Institute of Occupational HealthOslo, Norway; ^3^Department of Occupational Medicine, Haukeland University HospitalBergen, Norway; ^4^Department of Clinical Psychology, University of BergenBergen, Norway

**Keywords:** five-factor model, personality, bullying, observers, experiment

## Abstract

Previous studies on the personality of bullies and targets have exclusively been based on self-report. Against this backdrop we conducted a between group experimental vignette study with three conditions, describing a bully, a target and a control, respectively. Students (*n* = 242) were recruited as participants and rated the target on the observer rating version of the NEO Five Factor Inventory-Revised reflecting the personality dimensions Neuroticism, Extroversion, Openness to experience, Agreeableness and Conscientiousness. A MANOVA showed an overall significant effect of the experimental conditions. On Neuroticism significant differences between all conditions were found with targets rated highest and the control lowest. In terms of Extroversion the target was rated as lower than the control and the bully. No main effect was found for Openness. On Agreeableness the bully was rated as lower than both the target and the control. The bully was rated lower on Conscientiousness than the control. The significant differences reflected medium to large effect sizes. By and large the results are in agreement with comparable self-report data. The results are discussed in terms of practical implications and directions for future research are outlined.

## Introduction

Psychological research on bullying started to appear in the scientific literature during the 1970s (Olweus, 1978). Most of the early research focused on bullying among children in schools (Omoore and Hillery, 1989; Rigby and Slee, 1991; Boulton and Underwood, 1992; Whitney and Smith, 1993). Later on, emphasis was also put on adult bullying, especially occurring in the workplace (Leymann, 1990; Einarsen, 2000). Bullying in this context can be defined as “harassing, offending, socially excluding someone or negatively affecting someone’s work task. In order for the label bullying (or mobbing) to be applied to a particular activity, interaction or process it has to occur repeatedly and regularly (e.g., weekly) over a period of time (e.g., about 6 months). Bullying is an escalated process in the course of which the person confronted ends up in an inferior position and becomes the target of systematic negative social acts. A conflict cannot be called bullying if the incident is an isolated event or if two parties of approximately equal strength are in conflict” (Einarsen et al., 2003, p. 15).

Workplace bullying seems to be a widespread and common problem. A meta-analysis showed an average prevalence of 14.6% when employing targets’ self report, although the estimates varied significantly according to assessment method, sample and geographical location. Due to the significant negative consequences of workplace bullying in terms of reduced job satisfaction (Rodriguez-Munoz et al., 2009), ill-health (Kemp, 2014), and reduced productivity (McTernan et al., 2013) the need for more research into its antecedents has been emphasized by several scholars (Razzaghian and Shah, 2011; Samnani and Singh, 2012; Kemp, 2014). According to Samnani and Singh (2012) the causes of workplace bullying can be understood in terms of a hierarchical taxonomy ranging from individual factors such as personality (Linton and Power, 2013) to cultural/societal antecedents such as power distance (Vega and Comer, 2005) and masculinity-femininity (Lutgen-Sandvik et al., 2007).

Regarding personality as a potential antecedent for bullying the majority of previous research is based on the five-factor model of personality (Nielsen et al., 2017), probably due to the consistent cross-cultural validation of this model (McCrae et al., 1998). According to evolutionary perspectives the five-factor model of personality is closely linked to solving adaptive problems related to fundamental questions about human nature: Who will burden me with their problems and fail to cope well with adversity (Neuroticism)? Who will gain high status in the social hierarchy (Extroversion)? Who are able to provide good advice (Openness)? Who will be a good cooperator and reciprocator (Agreeableness)? Who will work industriously and dependably (Conscientiousness)? (Buss, 1991).

A meta-analysis of personality traits of bully and bullied among children and young adults (aged 8–25 years) showed that being a bully was positively related to Neuroticism and Extraversion, and inversely related to Openness, Agreeableness and Conscientiousness. Being a target was, however, only related (positively) to Neuroticism (Mitsopoulou and Giovazolias, 2015). In a recent meta-analysis of personality and workplace bullying it was shown that Extraversion, Agreeableness and Conscientiousness were all negatively associated with status as victim whereas victim status was positively associated with Neuroticism and unrelated to Openness. With exceptions of some studies on the dark triad personality traits (psychopathy, Machiavellianism and narcissism; Baughman et al., 2012) and interpersonal problems as personality dispositions (Glasø et al., 2009), very few studies have been conducted regarding personality traits of adult bullies. In a prison setting it was, however, found that status as a bully was inversely related to Agreeableness and positively associated with Neuroticism (Turner and Ireland, 2010). In another study bullies scored lower than non-bullies on Agreeableness, however, no group differences were found between bullies and non-bullies in regard to Conscientiousness, Extraversion and Neuroticism (Seigne et al., 2007).

A significant limitation of research into personality and bullying is that more or less all previous studies have relied on self-report of personality. Although such data may provide important insight into the personality of individuals, studies have shown that observer ratings of personality in specific contexts, such as the work environment, often do not correspond well with self-reported data (Mount et al., 1994). In addition, observers in most situations conduct dispositional attributions of others (Reeder et al., 2004). According to the five-factor model of personality inferences about the traits of others are important for adaptation to our social environment. The inferred or attributed traits will in line with this perspective guide our social interactions (Buss, 1991). For example, a person with high scores on Neuroticism may be avoided by others in order for the others not to be bothered with problems while a person with high scores on Extraversion can be assumed to be popular and may as such be approached in sociable situations. Knowledge about which traits observers attribute to targets and bullies is, however, sorely lacking, despite the fact that inferred traits may guide behavior toward them, as noted above. Moreover, the vast majority of research into bullying and dispositional traits is based on non-experimental studies preventing researchers from drawing causal inferences. However, by manipulating the roles as targets and bully and keeping other variables constant one can conclude more certainly about the impact of such roles in terms of trait attributions. Against this backdrop we designed an experimental vignette study investigating dispositional attributions made toward a bully, a target and a control person/condition. The research question addressed was the following: Which traits, in line with the five factor model of personality (Neuroticism, Extroversion, Openness, Agreeableness and Conscientiousness), will observers attribute to targets and bullies in a workplace, as compared to each other and controls, respectively?

## Materials and Methods

### Sample

The sample comprised 242 students, of whom 194 were females (80.2%) and 48 males (19.8%). They were recruited at lectures on two higher educational institutions in Bergen, Norway. The vast majority was undergraduate students in psychology. The mean age of the sample was 21.63 years (*SD* = 4.11).

### Instruments/Material

#### Demographics

The questionnaire contained items assessing participants’ age and gender.

#### The NEO Five Factor Inventory-Revised (NEO-FFI-R)

This scale contains 60 items and is a short version (McCrae and Costa, 2004) of the NEO-Personality Inventory Revised (NEO-PI-R) which have 240 items (Costa and McCrae, 1992). The NEO-PI-R was designed for assessing the five personality dimensions: Neuroticism, Extraversion, Openness to experience, Agreeableness and Conscientiousness as well as six sub-dimensions of each of these five main dimensions (Costa and McCrae, 1992). As a short version the NEO-FFI-R measures the five dimensions and does not provide scores for any sub-dimension (McCrae and Costa, 2004). In the present study an observer rating version of the NEO-FFI-R was administered (McCrae and Costa, 2004). NEO-FFI-R includes descriptive statements that participants respond to on a 5-point scale ranging from 0 (strongly disagree) to 4 (strongly agree). For each of the personality traits, a composite score was calculated by adding the participants’ ratings on the 12 corresponding items. A total of 28 NEO-FFI-R items are reverse-worded and was recoded accordingly. Possible total scores on each personality trait ranged from 0 to 48. In the present study the Cronbach’s alphas were 0.79 (Neuroticism), 0.78 (Extraversion), 0.65 (Openness to experience), 0.81 (Agreeableness) and 0.82 (Conscientiousness), respectively.

#### Descriptive Scenarios

A scenario was developed providing an outline of behavior and experiences of a target person (N) during a typical workday. The scenario was written so that two elements related to each of the five personality dimensions were incorporated. The two elements suggested for all dimensions both high (e.g., in terms of Neuroticism – afraid of dog) and low tendencies (e.g., in terms of Neuroticism – walked voluntarily in a cemetery in the dark) scores on the trait, respectively. This provided an ambiguous description with the aim of facilitating attributions related to the experimental conditions. Three different conditions depicting a bullied, a control, and a target, respectively, were inserted into the scenario: Condition 1 (Bullied): “They did not include N in the conversation and laughed contemptuously once she tried so say something. This was typical for such situations and for the working environment of N.” Condition 2 (Neutral): “Everybody around the table was included in the conversation, which was typical for those involved.” Condition 3 (Bully): “N and several of the colleagues excluded one of the colleagues at the table from the conversation and laughed contemptuously when she once tried to say something. It was typical that N treated the colleague in question like that.”

### Procedure

A questionnaire package containing an instruction (complete the questions about you, read the story and rate the person depicted on the 60 items listed on the following pages), one of the three versions of the storyline/scenarios and the observer-rating version of the NEO-FFI-R were administered. The packages were organized before the lectures in a consecutive order (one package containing story 1, 2, and 3 and then repeated in the same order). One questionnaire package was given to each student as they entered the lecture, hence a consecutive randomization procedure was used. Each participant responded to only one of the three scenarios. A total of 268 students were asked to participate of which 242 agreed (74 in the target, 88 in the control and 80 in the bully condition, respectively), yielding a response rate of 90.3%.

### Statistical Analyses

Statistical analyses were performed in SPSS, version 23.0. Univariate descriptive analyses of each study variable were conducted and results were calculated in terms of means and standard deviations or as percentages. A one-way ANOVA analysis was conducted in order to investigate if there were any age differences between the participants across the three conditions and a chi-square test was performed to detect potential gender differences across conditions. A MANOVA-analysis was then conducted in order to investigate whether there were significant differences in the mean scores of the five personality traits across the three conditions. *A priori* it was decided to use a one-way ANOVA for each personality dimension in the case of a significant overall MANOVA result. Bonferroni-correction would be used in cases of significant main effects of the ANOVAs. A power analysis was conducted based on the G^∗^Power software, version 3.1.7 (Faul et al., 2007). The analysis showed that for a MANOVA with three groups and five response variables, alpha at 0.05, power (1 – β) set to 0.80 and effect size (f^2^) set to 0.0625 (medium) a total of 135 subjects would be needed. In order to evaluate the difference in mean scores across the conditions in terms of effect sizes, Cohens *d* were calculated. As a benchmark effect sizes of 0.2, 0.5, and 0.8 are considered as small, medium, and large, respectively (Cohen, 1988).

## Results

There were no significant gender differences (*χ^2^* = 2.50, *df* = 2, *p* > 0.05) nor any age differences (*F*_2,239_ = 0.02, *p* > 0.05) between the participants across the three conditions (see **Table 1**). The result from the MANOVA was significant (*F*10,470 = 20.90, *p* < 0.001, *Wilk’s Λ* = 0.48). Accordingly, a one-way ANOVA with a Bonferroni *post hoc* test was performed for each of the five personality dimensions. The results are presented in **Table 2**. A significant main effect of experimental condition was found for Neuroticism. The mean score of all conditions differed significantly. The neutral condition had the lowest mean score whereas the target condition had the highest mean score. The effect sizes were medium (target vs. bully and neutral vs. bully) and large (target vs. neutral). A significant main effect of condition was also found for Extroversion, where the bullied condition had lower mean score than the two other conditions. Both these significant differences amounted to high effect sizes. No significant main effect of condition was found for Openness. The largest and significant main effect of the experimental condition was found for Agreeableness, where experimental condition explained one third of the variance. No difference was found between the target condition and the neutral condition. However, the mean score was significantly lower in the bully condition compared to the target condition and the neutral condition, both with large effect sizes. A significant main effect was also found for Conscientiousness where the mean score in the neutral condition was significantly higher (with a medium effect size) compared to the bully condition.

**Table 1:** Descriptive statistics in terms of age and gender of the participants across the three experimental conditions (*N* = 242).

	Condition 1 (Target; *n* = 74)	Condition 2 (Neutral; *n* = 88)	Condition 3 (Bully; *n* = 80)	Significance test
Age: mean (*SD*)	21.70 (3.87)	21.58 (3.80)	21.63 (4.66)	*F*_2,329_ = 0.02 *p* > 0.05
Gender (%)	25.7% 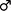	15.9% 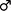	18.8% 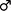	*χ^2^* = 2.50,
	74.3% 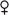	84.1% 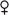	81.3% 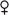	*p* > 0.05

**Table 2:** Means (M) and standard deviation (SD) and the results from the one-way ANOVAS regarding the effects of the target, neutral and bully condition on the five personality dimensions (*N* = 242).

	Target (*n* = 74)		Neutral (*n* = 88)		Bully (*n* = 80)		*F*_2,229_	*η^2^*	*Post hoc*^1^	Effect size a^2^	Effect size b^2^	Effect size c^2^
Personality dimension	*M*	*SD*	*M*	*SD*	*M*	*SD*						
Neuroticism	25.37	5.02	20.18	5.71	22.68	5.57	18.12^∗∗^	0.13	a,b,c	0.96	0.51	0.44
Extroversion	19.35	5.32	23.46	5.54	23.45	4.58	16.18^∗∗^	0.12	a,b	0.74	0.83	0.00
Openness	19.95	4.99	19.85	4.79	18.34	4.40	2.91	0.02		0.02	0.34	0.33
Agreeableness	27.27	4.55	27.31	5.19	19.69	5.67	57.78^∗∗^	0.33	b,c	0.01	1.47	1.40
Conscientiousness	33.30	5.29	33.76	5.94	31.04	6.19	5.09^∗∗^	0.04	c	0.08	0.39	0.63

*^∗^*p* < 0.05, ^∗∗^*p* < 0.01, (a) target compared to neutral, (b) target compared to bully, (c) neutral compared to bully, ^1^Significant group differences (Bonferroni corrrection), ^2^Cohens d, η^2^ = eta squared.*

## Discussion

The present study showed that observers rated the personality of targets and bullies differently from that of a control, both in terms of Neuroticism, Extraversion, Agreeableness and Conscientiousness. These findings may be seen in light of and have implications for knowledge on the actual personality of targets and bullies (Nielsen et al., 2017). Yet, the results may also be related to and have implications for knowledge on observers’ attribution of traits (see also Weber et al., 2013).

The highest effect size concerning the difference between target and control was found for Neuroticism. This finding is in line with meta-analytic data on studies among children and young adults (Mitsopoulou and Giovazolias, 2015) showing that Neuroticism was the only dimension that was significantly associated with self-reported victimization. The results are also in accord with similar studies on adult victims showing highest effect size for Neuroticism (Nielsen et al., 2017). Several factors may explain why victimization is associated with Neuroticism. One explanation relates to the assumption that Neuroticism is related to behaviors that may be regarded as annoying and bothersome by others, thereby triggering negative reactions from colleagues (Milam et al., 2009). It is also conceivable that targets of bullying become more anxiety-prone over time due to systematic and long-term exposure to bullying. This notion has been supported by longitudinal studies (Rodriguez-Munoz et al., 2015) albeit not consistently (Nielsen and Knardahl, 2015; Podsiadly and Gamian-Wilk, 2017). The findings are also in line with studies showing a tendency, especially among bullies, to morally justify and to attribute bulling behavior to negative traits of the victim (Thornberg and Jungert, 2014). In the present study the bully were also regarded as more neurotic than the control. This is in line with results from a study on adults prisoners (Turner and Ireland, 2010) and is also consistent with studies showing that Neuroticism is related to impulsive (angry, irritable or expressive) aggression (Gauthier et al., 2009).

On the Extroversion dimension the target was rated lower than both the bully and the control with large effect sizes. This is consistent with the results from the meta-analysis concerning victimization and personality among adults in the workplace (Nielsen et al., 2017).

Several scholars regard behaviors such as assertiveness and power display as central aspects of extraversion (Digman, 1990), which would render subjects low on this trait more susceptible to workplace bullying than those with higher scores. In addition, people with low scores on Extraversion often receive less social support (Swickert et al., 2002), which also may make them more likely than others to become targets of bullying. However, in an experimental study of cyberbullying it was shown that participants attributed more responsibility for a bullying incident to the victim when the victim was presented as extraverted than when the victim was presented as less extraverted (Weber et al., 2013). No significant differences between conditions were found for Openness. This is in line with the meta-analysis on children and young adults, the meta-analysis concerning victimization and personality among adults at work and a study of bullying among prisoners (Turner and Ireland, 2010). These findings and relevant trait descriptors such as Openness to feelings and to new ideas, flexibility of thought, and readiness to indulgence in fantasy (Digman, 1990) suggest that this personality dimension is of little relevance for bullying behavior. Another explanation to why the scores on Openness did not differ between the experimental conditions in the present study may relate to the relatively lower internal consistency found for this dimension compared to the other four dimensions. Also, it should be noted that the Openness dimension has been difficult to replicate across cultures, and therefore there is uncertainty about the validity of this specific dimension (John et al., 2008). Yet, quite another explanation is suggested by the findings in a study by Glasø et al. (2007) on the personality of self-reported targets of bullying, recruited among members of a Norwegian self-help association. Again, no relationship was found between being a target of bullying and Openness. Yet, a cluster analysis showed that behind this finding, two very different target groups existed on this dimension where one cluster of targets (one third) scored very low on Openness while a larger cluster (two thirds) scored very high on Openness. Hence, being a target may actually be related to extreme scores on this dimension, with reasonable theoretical explanations, as being low may be a risk of becoming a target due to ones lack of imagination, lack of humor and lack of intellectual resources, while being on the other end of the spectrum may render the person a victim of envy or sanctions for being an overachiever as compared to group norms.

The largest differences between our experimental conditions were found for the Agreeableness dimension, where the three conditions explained one third of the variance. Here the bully was rated as significantly lower than the target and the control, both contrasts equaling large effect sizes. These findings are in agreement with the meta-analysis on studies among children and young adults (Mitsopoulou and Giovazolias, 2015) and with results based on self-report of adults bullies (Seigne et al., 2007; Turner and Ireland, 2010). The results are further in line with the notion that low scores on Agreeableness involve preoccupation with one’s own goals and interests and a lack of sympathy for others suffering (Costa and McCrae, 1997). People scoring low on Agreeableness are typically less motivated than those with high scores to maintain positive interpersonal relationships, which also may explain why people low on Agreeableness are more inclined to act aggressively toward others (Gleason et al., 2004).

In terms of Conscientiousness the results from the present experiment showed that the bully was rated lower than the control. However, this finding was at odds with finding from the meta-analysis on studies among children and young adults that reported no association between bullying behavior and Conscientiousness (Mitsopoulou and Giovazolias, 2015). In addition, the finding from the present study concerning Conscientiousness is not supported by self-report data among adult bullies (Seigne et al., 2007; Turner and Ireland, 2010). However, the findings are in line with research showing that individuals with antisocial personality score relatively low on Conscientiousness (Miller and Lynam, 2001).

Taken together the personality attributions made by observers in this vignette based experiment fit well with self-reported personality traits of bully and target and are also by and large in line with relevant theoretical perspectives on the relationship between personality and bullying. As such the study supports the notion of the general observer as a social scientist in terms of attribution (Kelly, 1973).

### Practical Implications

In terms of practical implications the results overall indicate that those who observe targets of bullying in an organization seem to infer that such persons relatively speaking are neurotic and introverted. Although co-workers, e.g., through anti-bulling policies, normally are encouraged to support targets (Murray, 2009) the current study suggests that observers may be inclined rather to avoid the targets in accordance with evolutionary perspectives of the traits inferred (Buss, 1991). The bully was rated as relatively low on Agreeableness and Conscientiousness. Bullies may induce fear in coworkers and as such force them to act in certain ways (Georgakopoulos et al., 2011). Still, they will overall, and in line with the results from the present study, typically not be regarded as good cooperators and reciprocators or as someone who will work industriously and dependably (Buss, 1991). This can be assumed to lower the trust in the organization. The findings indicating that observers more or less accurately will tend to see bullies as being low on Conscientiousness, may influence how others, e.g., managers, will handle a given case of bullying and the involved employees, not trusting the bully to behave responsibly in the future, again lower the trust in the involved parties.

The overall “take-home message” from the present study in terms of implications is that trait inferences regarding bullying should be taken into consideration in terms of workplace countermeasures against bullying.

### Limitations and Strengths

Some limitations of the present study should be mentioned. We cannot rule out that some participants were able to infer the real aim of the study, hence the results could thus have been influenced by demand characteristics (Orne, 1962). However, an independent measure design was used explicitly in order to avoid this (Coolican, 2014). The vignette presented can arguably be regarded as artificial which may have influenced the findings. A meta-analysis comparing laboratory and naturalistic experiments concluded, however, that results from the different settings overall were converging (Anderson et al., 1999). The participants in the present study were students with a female preponderance, hence the results cannot as such be generalized to other populations without reservations. The target depicted in the vignettes was female which may also limit the generalizability. In addition, the type of bullying depicted was rather narrow and the results might have been different if other bullying acts (Einarsen et al., 2009) had been incorporated in the vignette. In terms of assets of the present study the use of an experimental design deserve mention as this ensured conclusions about the factors that causally contributed to the specific trait attributions. Still, it should be noted that the findings of this study do not provide information about the causal order of the associations between bullying (be it perpetrated or experienced) and personality. Hence, the study cannot be used to draw conclusion about whether certain personality characteristics increase the risk of being bullied/bullying others or whether bullying influence the personality characteristics of those involved (for longitudinal findings, see Nielsen and Knardahl, 2015; Podsiadly and Gamian-Wilk, 2017). The sample size was predetermined based on *a priori* power calculation. The bullying behavior described in the vignette adhered specifically to several of the defining elements of bulling (e.g., duration; Einarsen et al., 2003). Although experimental studies into other effects of bullying (e.g., psychological contract breach) has been conducted (Kakarika et al., 2017) the present study is to the best of our knowledge the first that has employed an experimental design to investigate how targets and bullies are perceived in terms of personality and contributes as such with new knowledge as well as with a novel methodological approach to the field of bullying research.

## Conclusions and Directions of Future Research

The findings of this study confirms previous findings from self-report studies by showing that targets of bulling typically score relatively high on Neuroticism and low on Extroversion (Nielsen et al., 2017). The results from the present study are furthermore in line with self-report studies where bullies report relatively low scores on Agreeableness and Conscientiousness (Seigne et al., 2007; Turner and Ireland, 2010; Mitsopoulou and Giovazolias, 2015).

Using the same methodological approach as in the present study future research should aim to investigate how different aspects of bullying (e.g., verbal vs. physical, characteristics of victim and perpetrator) influence trait attributions. Future research should also investigate a wider set of personality attributes than those employed in the present study. Research should also aim to widen the scope of reactions of observers beyond mere personality attributions and should as such, based on experimental approaches, elucidate emotional reactions (including physiological reactions), behavioral inclinations and organizational outcomes to bulling behaviors. Furthermore, other study groups than students should be invited as observers with the possibility of increasing the generalizability of the findings. As studies have shown that short-term interventions may reduce self-blame among targets (Boulton and Boulton, 2017), future studies should also investigate effects of interventions aiming to modify blame and attributions related to bullying in general. Targets emotional reactions to bullying have been shown to modify attributions of blame by teachers toward victims as well as bullies (Sokol et al., 2016). Futures studies should accordingly investigate how targets reactions impact attributions of bulling behavior within the workplace. Finally, previous findings have shown that a person’s own exposure to bullying color his/her witness report of other’s exposure to bullying (Nielsen and Einarsen, 2013). To rule out the impact of this kind of bias, upcoming studies should adjust for the respondents own personality characteristics as well as their own experience with bullying.

## Ethics Statement

The study was approved by the Institutional Review Board at the Faculty of Psychology, University of Bergen. Consent was deemed provided upon completion of the questionnaire.

## Author Contributions

SP contributed to the conception and design of the work, the acquisition, analysis, and interpretation of data. MN, NM, CA, and SE contributed to the interpretation of data for the work. SP drafted the work. All authors revised the work critically in terms of important intellectual content. All authors approved the final version and are accountable for all aspects of the work in terms of ensuring that questions related to the accuracy or integrity of any part of the work were appropriately investigated and resolved.

## Conflict of Interest Statement

The authors declare that the research was conducted in the absence of any commercial or financial relationships that could be construed as a potential conflict of interest.
